# Age and Sex Affect Essential Tremor (ET) Plus: Clinical Heterogeneity in ET Based on the National Survey in China

**DOI:** 10.14336/AD.2022.1205

**Published:** 2023-08-01

**Authors:** Qiying Sun, Runcheng He, Hongyan Huang, Hongmei Cao, Xuejing Wang, Hong Liu, Chunyu Wang, Lifang Lei, Puqing Wang, Guiyun Cui, Jianjun Ma, Ping Gu, Di An, Min Jia, Zhanfang Sun, Heng Wu, Jinsheng Lin, Jiayu Tang, Xun Zhou, Mingqiang Li, Sheng Zeng, Yase Chen, Xinxiang Yan, Jifeng Guo, Qian Xu, Zhenhua Liu, Lu Shen, Hong Jiang, Xinyin Wu, Qin Xiao, Haibo Chen, Yanming Xu, Beisha Tang, the China Essential Tremor Alliance

**Affiliations:** ^1^Department of Neurology, Department of Geriatric Neurology, Xiangya Hospital, Central South University, Changsha, Hunan, China.; ^2^National Clinical Research Center for Geriatric Disorders, Xiangya Hospital, Central South University, Changsha, Hunan, China.; ^3^Department of Neurology, West China Hospital, Sichuan University, Chengdu, China.; ^4^Department of Neurology, The First Affiliated Hospital of Xi'an Jiaotong University, Xi'an, Shaanxi, China.; ^5^Department of Neurology, The First Affiliated Hospital of Zhengzhou University, Zhengzhou, Henan, China.; ^6^Department of Neurology, Heping Hospital Affiliated to Changzhi Medical College, Changzhi, Shangxi, China.; ^7^Department of Neurology, The Second Xiangya Hospital, Central South University, Changsha, Hunan, China.; ^8^Department of Neurology, The Third Xiangya Hospital, Central South University, Changsha, Hunan, China.; ^9^Department of Neurology, Xiang Yang No. 1 People’s Hospital Affiliated to Hubei University of Medicine, Xiangyang, Hubei, China.; ^10^Department of Neurology, The Affiliated Hospital of Xuzhou Medical University, Xuzhou, Jiangsu, China.; ^11^Department of Neurology, Henan Provincial People's Hospital, Zhengzhou, Henan, China.; ^12^Department of Neurology, The First Hospital of Hebei Medical University, Shijiazhuang, China.; ^13^Department of Neurology, Affiliated Hospital of Hebei University, Baoding, Hebei, China.; ^14^Department of Neurology, The Central Hospital of Enshi Tujia and Miao Autonomous Prefecture, Enshi, Hubei, China.; ^15^Department of Neurology, Shandong Provincial Hospital Affiliated to Shandong First Medical University, Jinan, Shandong, China.; ^16^Department of Neurology, The First Affiliated Hospital of University of South China, Hengyang, Hunan, China.; ^17^Department of Neurology, Xiangtan Central Hospital, Xiangtan, Hunan, China.; ^18^Department of Neurology, Hunan Provincial Brain Hospital, Changsha, Hunan, China.; ^19^Department of Geriatric Neurology, The Second Xiangya Hospital, Central South University, Changsha, Hunan, China.; ^20^Department of Epidemiology and Health Statistics, Xiangya School of Public Health, Central South University, Changsha, Hunan, China.; ^21^Department of Neurology and Institute of Neurology, Ruijin Hospital, Shanghai Jiao Tong University School of Medicine, Shanghai, China.; ^22^Department of Neurology, Beijing Hospital, National Center of Gerontology, Beijing, China.; ^23^Key Laboratory of Hunan Province in Neurodegenerative Disorders, Central South University, Changsha, Hunan, China.

**Keywords:** essential tremor, essential tremor plus, the national survey of essential tremor plus in china, registry study, clinical heterogeneity

## Abstract

The new term essential tremor (ET) plus was proposed in the 2018 tremor consensus criteria. The National Survey of Essential Tremor Plus in China, a large multicenter registry study, aimed to evaluate the clinical features of pure ET and ET plus and explore possible factors related to ET plus. All patients with ET underwent neurological examination and neuropsychological assessment at 17 clinical sites. The diagnosis was made according to the 2018 consensus criteria. Clinicodemographic characteristics were analyzed. A total of 1160 patients were included, including 546 patients with pure ET and 614 patients with ET plus. The proportion of females was significantly higher in the ET plus than that in the pure ET (P = 0.001). The age at onset (AAO) of pure ET showed a bimodal distribution, with peaks in the 2nd and 5th decades. However, the AAO of the ET plus group demonstrated a skewed distribution, with a single peak in the 6th decade. Female sex (OR=1.645, P<0.001), older age (OR=1.023, P<0.001), lower educational level (OR=0.934, P<0.001), head tremor (OR=1.457, P<0.001), and higher the Tremor Research Group Essential Tremor Rating Assessment Scale (TETRAS)-II scores (OR=1.134, P<0.001) were significantly associated with ET plus. Old age and female sex may contribute to ET plus development. Pure ET showed a bimodal distribution for AAO, whereas ET plus showed a unimodal distribution. It remains unclear whether pure ET and ET plus are merely different stages of a single disease or represent distinct disease entities.

## INTRODUCTION

Essential tremor (ET) is one of the most common movement disorders, with a worldwide prevalence of approximately 0.9% in the general population. The prevalence of ET increases progressively with advancing age, affecting approximately 5.79% of individuals aged > 65 years [1,2]. ET is familial in more than half of the patients with a typical autosomal dominant pattern. The 2018 consensus statement on tremors by the International Parkinson and Movement Disorder Society (IPMDS) defined ET as a pure tremor syndrome in which patients display action tremors of the bilateral upper limbs for at least three years, with or without a tremor in other locations (e.g., head, face, voice, or lower limbs). Additionally, the 2018 consensus statement proposed a new concept of ET plus, defined as ET in the presence of additional neurological signs of uncertain significance (e.g., impaired tandem gait, questionable dystonic posturing, mild memory impairment, or other mild neurological signs of unknown significance) [3].

This new clinic-based classification has generated interest and controversy [4]. A new classification approach can distinguish between patients with limited and typical clinical features and those with additional neurological signs. According to this new approach, many patients traditionally diagnosed with ET should be reclassified as either pure ET or ET plus under the new terminology [5,6]. Despite the significant clinical heterogeneity of ET, a recent postmortem study found no pathological differences in the cerebellum between ET and ET plus, suggesting no pathological differences between the two subtypes [7]. In contrast, several studies suggested that patients diagnosed with ET plus might not have ET but a distinct syndrome [8-9]. Other pathological studies were not uniform across ET patients [10], suggesting that ET was not a single disease. Previous findings have engendered great controversy regarding the categories of ET and ET plus. However, some previous studies retrospectively reclassified ET patients evaluated before 2018, which were prone to recall bias [11-14]. Several ET cohorts with relatively small sample sizes were prospectively enrolled, most of which were conducted in a single center [5, 15-20]. The Italian tremor network (TITAN) prospectively assessesed the phenomenology and natural history of different tremor syndromes based on the new classification in Italy [20]. To further study the clinical heterogeneity of ET according to the new consensus statement, determine the proportion of ET plus in mainland China, recognize the deviations from ET, evaluate the non-motor symptoms in patients with ET, and explore the possible factors related to ET plus, the China Essential Tremor Alliance launched a large multicenter registry study of ET named the National Survey of Essential Tremor Plus in China (NSETP-China).

## MATERIAL AND METHODS

### Study Design and Patients

**Figure 1.**
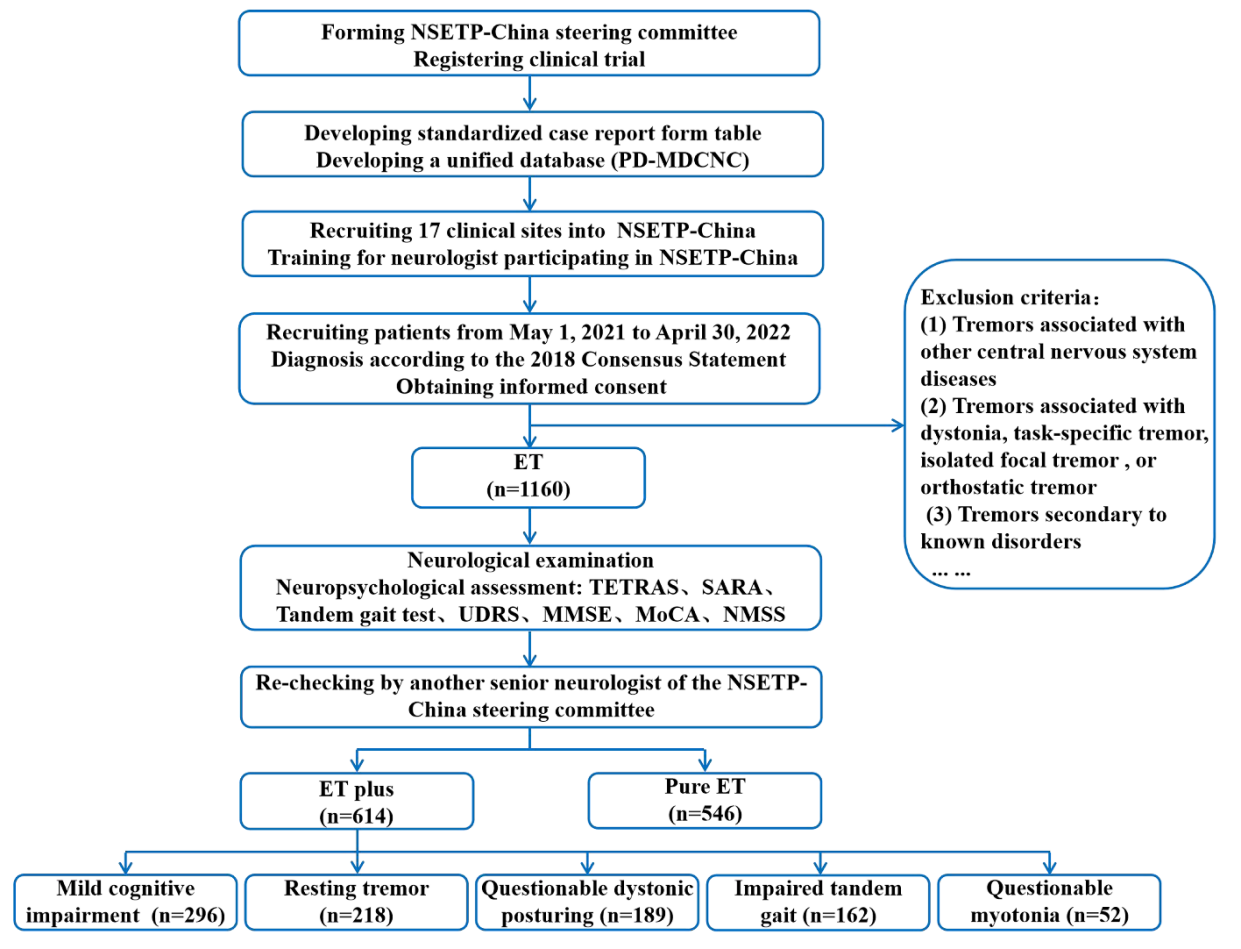
Flowchart of the National Survey of Essential Tremor Plus in China (NSETP-China).

The NSETP-China study was a nationwide, cross-sectional, multicenter registry cohort study. The study recruited both inpatients and outpatients from the Department of Neurology at 17 clinical sites between May 1, 2021, and April 30, 2022. The NSETP-China Steering Committee developed a unified database, a standardized case report form table, and a workbook. All neurologists participating in the clinical evaluation had undergone at least three training sessions with the NSETP-China steering committee to ensure the standardization of data acquisition (Fig. 1). All patients were diagnosed by at least two experienced neurologists according to the 2018 International Movement Disorder Society Tremor Group essential tremor diagnostic criteria [3] and were re-checked by another senior neurologist in the NSETP-China steering committee. Patients were excluded from the study if they had one of the following: (1) tremors associated with other central nervous system diseases, such as Parkinson’s disease (PD), Wilson’s disease, encephalitis, or stroke; (2) tremors associated with dystonic tremor, tremor associated with dystonia, task-specific tremor, isolated focal tremor (including isolated vocal tremor, isolated head tremor), or orthostatic tremor; or (3) other tremors secondary to known disorders, such as hyperthyroidism, sympathomimetic drugs, and pheochromocytoma. An age at onset (AAO) cutoff of ≥ 60 years was used to define “late-onset” ET.

This study was approved by the Medical Ethics Committee of Xiangya Hospital, Central South University and was conducted according to the principles of the Declaration of Helsinki. This study was registered at ClinicalTrials.gov (Identifier: NCT04837079). Informed consent was obtained from all patients.

### Assessments

Clinicodemographic data including sex, age, lifestyle, family history, AAO, disease duration, prescribed medications, medical history, motor symptoms, and non-motor symptoms were collected from the Parkinson’s Disease and Movement Disorders Multicenter Database and Collaborative Network in China (PD-MDCNC, http://pd-mdcnc.com) [21]. AAO was defined as the date of appearance of the first tremor. All patients underwent neurological examination and neuropsychological assessment. Tremor severity was evaluated with the Tremor Research Group Essential Tremor Rating Assessment Scale (TETRAS) [22]. TETRAS-I (items 1-12) was used to evaluate the impact of tremors on daily living on a scale of 0-4, whereas TETRAS-II (items 1-9) was used to evaluate tremor types (including postural and kinetic tremor), distribution (including head, face, voice, limbs, and trunk tremors), and severity. Physicians estimated the maximum amplitude of tremors and assigned corresponding rating scores (0-4), with higher scores indicating more severe tremors. Tremor asymmetry was defined as a difference of > 1 point in the total limb tremor scores (items 4, 5, 6, and 8) between the dominant and non-dominant arms.

**Table 1:** Comparison of demographic and clinical characteristics between the pure ET and ET plus.

Items	Pure ET (n=546)	ET plus (n=614)	*P*-value
**Sex (male %)**	323 (59.2%)	264 (43.0%)	**<0.001**
**Age (y)**	50.09±16.50	59.31±14.72	**<0.001**
**AAO (y)**	40.56±17.51	47.32±16.51	**<0.001**
**Duration (y)**	9.53±8.41	11.99±10.63	**<0.001**
**Family history (%)**	272 (49.8%)	297 (48.4%)	0.623
**Alcohol sensitivity (%)**	124/304 (40.8%)	139/308 (45.1%)	0.278
**Smoking (%)**	142 (26.0%)	128 (20.8%)	**0.038**
**Alcohol consumption (%)**	109 (20.0%)	105 (17.1%)	0.210
**Hypertension**	83 (15.2%)	158 (25.7%)	**<0.001**
**Diabetes mellitus**	28 (5.1%)	67 (10.9%)	**<0.001**
**Hyperlipidemia**	30 (5.5%)	37 (6.0%)	0.698
**Tremor distribution**			
**Head (%)**	143 (26.2%)	227 (37.0%)	**<0.001**
**Face (%)**	104 (17.9%)	163 (22.9%)	**0.002**
**Voice (%)**	117 (21.4%)	182 (29.6%)	**0.001**
**Upper limbs (%)**	546(100%)	614 (100%)	-
**Lower limbs (%)**	140 (25.6%)	190 (30.9%)	**0.046**
**Tremor types**			
**Posture tremor (%)**	546 (100.0%)	614 (100%)	-
**Kinetic tremor (%)**	518 (94.9%)	591 (96.3%)	0.252
**Intention tremor (%)**	158 (28.9%)	262 (42.7%)	**<0.001**
**Tremor asymmetry**	185 (33.9%)	216 (35.2%)	0.643
**Tremor severity**			
**TETRAS-I**	11.17±8.99	15.48±10.12	**<0.001**
**TETRAS-II**	16.39±7.42	19.74±8.68	**0.003**
**NMSS**			
**Cardiovascular**	0.26±0.82	0.56±1.61	**<0.001**
**Sleep/fatigue**	2.98±4.42	4.22±5.17	**<0.001**
**Mood/cognition**	1.82±3.97	2.97±5.96	**<0.001**
**Perceptual problems**	0.10±0.47	0.23±0.81	**0.002**
**Attention/memory**	1.23±2.06	2.96±4.06	**<0.001**
**Gastrointestinal symptoms**	0.53±1.62	1.06±2.31	**<0.001**
**Urinary symptoms**	1.05±2.47	2.02±3.74	**<0.001**
**Sexual function**	0.21±1.08	0.34±1.53	0.097
**Other symptoms**	0.72±1.96	1.28±2.82	**<0.001**
**NMSS total score**	8.91±11.52	15.73±16.94	**<0.001**

Data for continuous variables are presented as mean ± standard deviation. Values in bold refer to statistically significant differences (*P* < 0.05). Abbreviations: ET, essential tremor; y, years; AAO, age at onset; TETRAS, Tremor Research Group Essential Tremor Rating Assessment Scale; NMSS, Non-Motor Symptoms Scale.

Ataxia severity was assessed using the Scale for the Assessment and Rating of Ataxia (SARA) [23]. Tandem gait was assessed by asking the patients to take 10 consecutive tandem steps along a straight line while keeping their arms at their sides. Patients were allowed to complete two attempts serially; missteps were recorded at every turn, and the best performance was recorded [24]. Dystonic posturing was assessed using the Unified Dystonia Rating Scale (UDRS) [25]. The Non-Motor Symptoms Scale (NMSS) was used to evaluate the severity of non-motor symptoms. The Mini-Mental State Examination (MMSE) and Montreal Cognitive Assessment (MoCA) were used to measure global cognitive function [26,27].

The NSETP-China website was an essential component of the study design. All study data were integrated into the NSETP-China study database using the PD-MDCNC.

### Assigning ET plus Diagnoses

ET plus was defined using the new consensus criteria, namely ET with any of the following neurological soft signs: (1) mild cognitive impairment, (2) questionable dystonic posturing, (3) impaired tandem gait, (4) resting tremor, and (5) questionable myotonia. Mild cognitive impairment was defined as any of the following: (1) MMSE total score < 17 for illiterate, < 20 for elementary education, or < 24 for middle school education or above; (2) MoCA total score < 26 (MoCA < 25 for participants with ≤ 12 years of education) [5,28]. Questionable dystonic posturing was defined as a UDRS score ≥ 1. Impaired tandem gait was defined as at least two missteps out of a 10-step trial. The resting tremor was assessed while participants stood with their arms relaxed and at rest on their sides, or while seated with their hands on their laps and forearm supported against gravity [29,30]. Questionable myotonia was established by neurological examination.

### Statistical Analysis

The data were coded, cleaned, and checked for completeness. Continuous variables were described as mean ± standard deviation, and categorical variables were described as percentages and frequencies. The Kolmogorov-Smirnov (K-S) test was used to evaluate the normal distribution of the variables. An independent samples t-test was used for continuous variables that were normally distributed. The Mann-Whitney test was used if the continuous variables were not normally distributed. Binary variables were compared using the chi-squared test. Binary logistic regression analysis was used to identify the significant factors associated with ET plus. To adjust for age and AAO, we performed linear or logistic regression to compare the clinical features between female and male ET patients. To adjust for sex and disease duration, we performed linear or logistic regression to compare the clinical features between late-onset ET and ET patients with AAO < 60 years. All statistical analyses were performed using SPSS version 25.0 (IBM Corp., Armonk, NY, USA). Statistical significance was set at *P <* 0.05.

**Figure 2.**
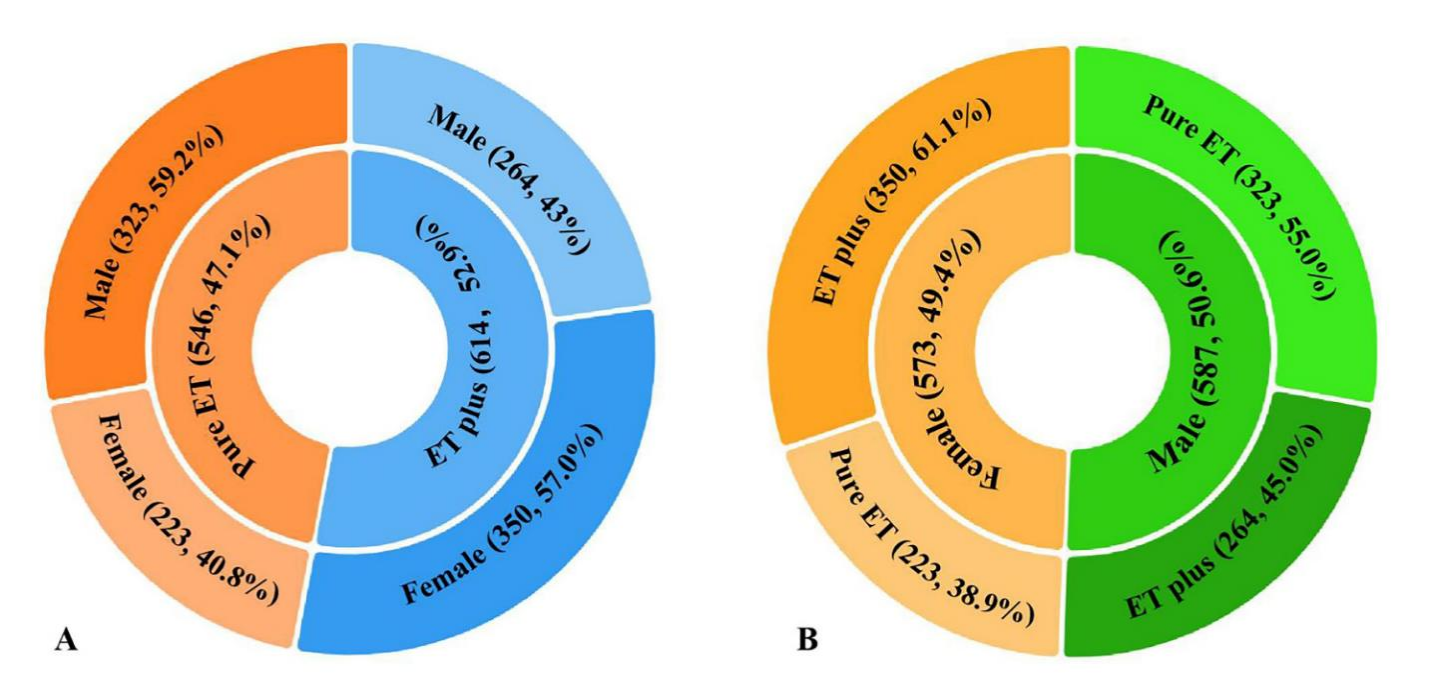
**Sex-related differences in the clinical phenotype**. (**A**) The proportion of female patients is higher in the ET plus group (57.0%) than that in the pure ET group (40.8%). (**B**) The proportion of ET plus is higher in females (61.1%) than that in males (45.0%).

## RESULTS

### Patient characteristics

**Figure 3.**
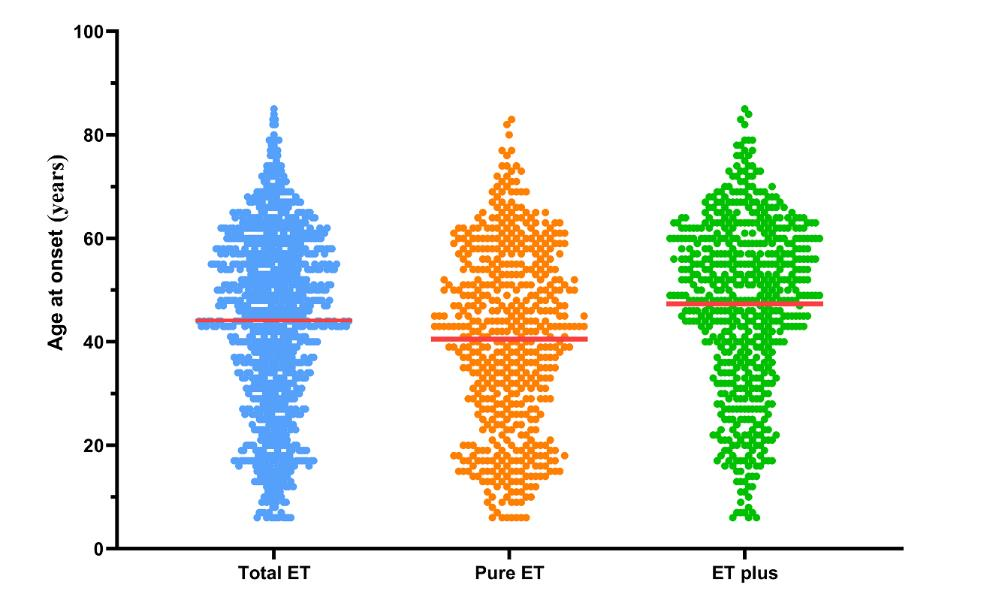
**Age at onset (AAO) distribution in total ET, pure ET, and ET plus**. The AAO of total ET patients (n=1160) shows a bimodal distribution with peaks in the 2^nd^ and ^6th^ decades of life. The AAO of the pure ET group (n=546) shows a bimodal distribution with peaks in the 2^nd^ and 5^th^ decades of life. The AAO of the ET plus group (n=614) demonstrates a skewed distribution with a single peak in the 6^th^ decade.

**Figure 4.**
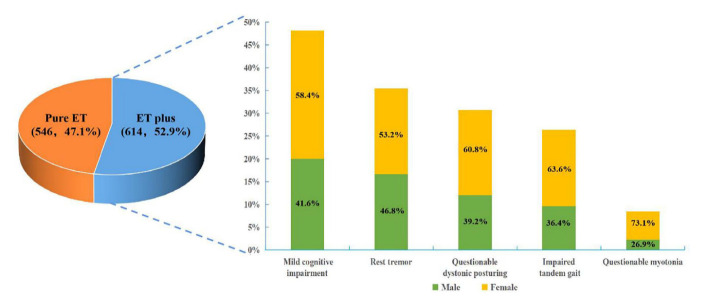
**ET plus (n=614) is more common than pure ET (n=546)**. Mild cognitive impairment (48.2%) is the most prevalent neurological soft sign in ET plus, followed by resting tremor (35.5%), questionable dystonic posturing (30.8%), impaired tandem gait (26.4%), and questionable myotonia (26.4%). A higher percentage of the female is found in each ET plus subgroup with various neurological soft signs.

A total of 1160 patients, including 587 (50.6%) males and 573 (49.4%) females, were recruited. Among them, 546 (47.1%) patients were classified as having pure ET, while 614 (52.9%) patients were classified as having ET plus. The clinicopathological characteristics of the patients were shown in Table 1. The proportion of female patients was significantly higher in the ET plus group than in the pure ET group (57.0% vs. 40.8%; X^2^ = 30.196, *P* < 0.001) (Fig. 2A). The proportion of ET plus was also significantly higher in females than in males (61.1% vs. 45.0%; X^2^ = 30.196, *P* < 0.001) (Fig. 2B). The mean age upon enrollment in ET plus group was significantly older (59.31±14.72 years vs 50.09±16.50 years; Mann-Whitney U test, *P* < 0.001). The AAO of all recruited patients showed a bimodal distribution, peaking at the 2^nd^ and 6^th^ decades of life. Similar to the AAO of all patients, the AAO of the pure ET group also showed a bimodal distribution, with peaks in the 2^nd^ and 5^th^ decades of life. However, the AAO of the ET plus group demonstrated a skewed distribution, with a single peak in the 6^th^ decade. The mean AAO of the pure ET group was significantly younger than that of the ET plus group (47.32 ± 16.51 years vs 40.56 ± 17.51 years; Mann-Whitney U test, *P* < 0.001) (Fig. 3). The tremor duration of the ET plus group was significantly longer than that of the pure ET group (11.99 ± 10.63 years vs 9.53 ± 8.41 years; Mann-Whitney U test, *P* < 0.001). In the ET plus group, 296 (48.2%) patients had mild cognitive impairment, 218 (35.5%) patients had resting tremor, 189 (30.8%) patients had questionable dystonic posturing, 162 (26.4%) patients had impaired tandem gait, 52 (8.4%) patients had questionable myotonia on examination, and 234 (38.1%) patients had multiple soft neurological signs (Fig. 4).

Meanwhile, there was no significant group difference with respect to a family history of tremors (49.8% vs. 48.4%; X^2^ = 0.242, *P* = 0.623) or alcohol sensitivity (40.8% vs. 45.1%; X^2^ = 1.176, *P* = 0.278). Smoking rates were higher in the ET plus group than in the ET group with statistical significance (26.0% vs. 20.8%; X^2^ = 4.310, *P* = 0.038). Of the 1160 patients, only 206 (17.8%) were prescribed medication for tremors. Of the 206 treated patients, 122 (57.9%) and 63 (30.6%) patients had taken arotinolol and propranolol, respectively, and only 3 of the 206 (1.5%) patients had taken primidone. The proportion of patients who took tremor medications was significantly higher in the ET plus group than in the pure ET group (43.5% vs. 13.2%; X^2^ = 128.268, P < 0.001).

**Figure 5.**
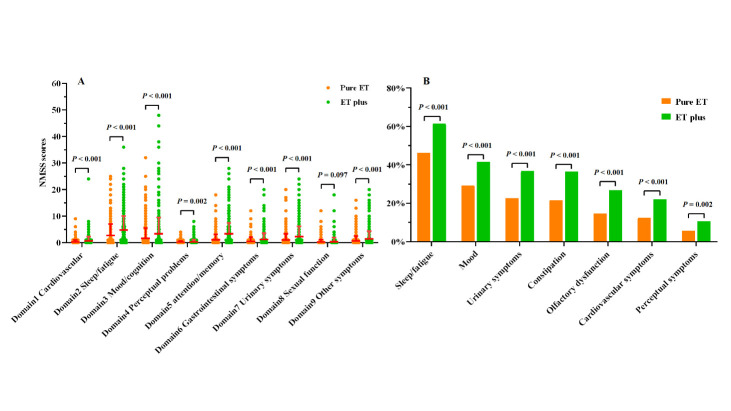
**Comparison of non-motor features between pure ET and ET plus**. (**A**) The NMSS sub-scores in the ET plus group were higher than those in the pure ET group (mean ± SD). (**B**) The frequency of non-motor symptoms presented in the ET plus group were higher than in the pure ET group. NMSS, Non-Motor Symptoms Scale.

### Features of motor and non-motor symptoms

Postural tremors were present in all patients with ET. A total of 518 (94.9%) patients in the pure ET group and 591 (96.3%) in the ET plus group showed both postural and kinetic tremors. Intention tremors were more common in the ET plus group than in the pure ET group reaching statistical significance (42.7% vs. 28.9%; X^2^ = 23.599, *P* < 0.001). Asymmetric tremors were present in 33.9% (185) of the pure ET group and 35.2% (216) of the ET plus group (X^2^ = 0.215, *P* = 0.643). A total of 378 (69.2%) patients in the pure ET group and 456 (74.3%) in the ET plus group reported increased severity of tremor symptoms since onset (X^2^ = 3.628, *P* = 0.057). Among all ET patients, 370 (31.9%) had head tremors, 330 (28.4%) had lower limb tremors, 299 (25.8%) had voice tremors, and 267 (23.0%) had facial tremors. The location of tremors varied, with a higher prevalence in the head, lower limbs, voice, and face in the ET plus group than in the pure ET group (X^2^ = 15.461, *P <* 0.001; X^2^ = 3.994, *P* = 0.046; X^2^ =10.190, *P* = 0.001; and X^2^ = 9.173, *P* = 0.002, respectively). TETRAS-I and TETRAS-II scores were significantly higher in the ET plus group than in the pure ET group (Mann-Whitney U test, all *P* < 0.001).

**Table 2:** Binary logistic regression analysis of factors associated with pure ET and ET plus.

Variables	Univariate P-value*	Multivariate OR (95% CI)	P-value
**Sex (female)**	<0.001	1.645	**<0.001**
**Age (y)**	<0.001	1.023	**<0.001**
**AAO (y)**	<0.001	-	-
**Duration (y)**	<0.001	-	-
**Education level**	<0.001	0.934	**<0.001**
**Family history of tremor (%)**	0.988	-	Not included
**Smoking (%)**	0.023	-	-
**Alcohol consumption (%)**	0.143		Not included
**Hypertension**	<0.001	-	-
**Diabetes mellitus**	<0.001	-	-
**Hyperlipidemia**	0.761	-	Not included
**Head tremor**	<0.001	1.457	**0.008**
**Face tremor**	0.003	-	-
**Voice tremor**	0.001	-	-
**Lower limbs tremor**	0.046	-	-
**TETRAS-I**	<0.001	-	-
**TETRAS-II**	<0.001	1.032	**<0.001**

Values in bold refer to statistically significant differences (*P* < 0.05). Abbreviations: ET, essential tremor; y, years; AAO, age at onset; TETRAS, Tremor Research Group Essential Tremor Rating Assessment Scale; NMSS, Non-Motor Symptoms Scale.

**Table 3:** Comparison of demographic and clinical characteristics between ET patients with AAO below and above 60 years of age.

Items	AAO < 60 years (n=947)	AAO ≥ 60 years (n=213)	*P*-value
**Age (y)**	51.22±15.42	71.66±6.17	**<0.001**
**AAO (y)**	39.02±14.76	66.9±5.40	**<0.001**
**Sex (male %)**	496 (52.4%)	91 (42.7%)	**0.011**
**Duration (y)**	12.19±10.16	4.76±3.33	**<0.001**
**Family history (%)**	483 (51.0%)	86 (40.4%)	**0.005**
**Alcohol sensitivity (%)**	206/450 (45.8%)	17/82 (20.7%)	<0.001
**Tremor distribution**			
**Head (%)**	302 (31.9%)	68 (31.9%)	0.385^#^
**Face (%)**	201(21.2%)	66 (31.0%)	**<0.001^#^**
**Voice (%)**	237 (25.0%)	62 (29.1%)	**0.001^#^**
**Upper limbs (%)**	947 (100%)	213 (100%)	-
**Lower limbs (%)**	265 (28.0%)	65 (30.5%)	**0.015^#^**
**Tremor types**			
**Posture tremor (%)**	947 (100%)	213 (100%)	-
**Kinetic tremor (%)**	902 (95.2%)	207 (97.2%)	**0.009^#^**
**Intention tremor (%)**	344 (36.3%)	76 (35.7%)	0.103^#^
**Tremor severity**			
**TETRAS-I**	13.13±9.89	14.89±9.51	**<0.001^#^**
**TETRAS-II**	17.97±8.27	19.02±8.26	**<0.001^#^**
**Tremor asymmetry**	320 (33.8%)	81 (38.0%)	0.153^#^
**ET plus**	478(50.5%)	136(63.8%)	**<0.001^#^**
**Mild cognitive impairment**	216(22.8%)	80 (37.6%)	**<0.001^#^**
**Rest tremor**	178(18.8%)	40 (18.8%)	0.369^#^
**Questionable dystonic posturing**	155(16.4%)	34 (16.0%)	0.760^#^
**Impaired tandem gait**	120(10.1%)	42 (18.0%)	**<0.001^#^**
**Questionable myotonia**	35(3.7%)	17 (8.0%)	**0.010^#^**
**NMSS**	12.38±15.30	14.18±14.34	**<0.001^#^**

Data for continuous variables are presented as mean ± standard deviation. *P* value ^#^ is from linear or logistic regression and adjusted with variables of sex and disease duration. Values in bold refer to statistically significant differences (*P* < 0.05). Abbreviations: ET, essential tremor; y, years; AAO, age at onset; TETRAS, Tremor Research Group Essential Tremor Rating Assessment Scale; NMSS, Non-Motor Symptoms Scale

The NMSS sub-scores (except the sexual function sub-score) and NMSS total scores were significantly higher in the ET plus group (Mann-Whitney U test, all *P* < 0.05) (Figure 5A). Consistently, non-motor symptoms occurred more frequently in the ET plus group than in the pure ET group, with statistical significance (83.6% vs. 66.5%; X^2^ = 45.531, *P* < 0.001). In addition, significantly more patients in the ET plus group reported sleep/fatigue (61.4% vs. 46.3%; X^2^ = 26.428, *P* < 0.001), mood/apathy (41.5% vs. 29.1%; X^2^ = 19.392, *P* < 0.001), urinary symptoms (36.8% vs. 22.7%; X^2^ = 27.260, *P* < 0.001), constipation (36.5% vs. 21.4%; X^2^ = 31.554, *P* < 0.001), olfactory dysfunction (26.9% vs. 14.7%; X^2^ = 25.908, *P* < 0.001), cardiovascular symptoms (22.0% vs. 12.5%; X^2^ = 18.191, *P* < 0.001), and perceptual symptoms (10.6% vs. 5.7%; X^2^ =9.173, *P* = 0.002) (Fig. 5B).

### Risk factors of ET plus

Binary logistic regression analysis indicated that female sex (odds ratio (OR) = 1.645, *P* < 0.001), older age (OR = 1.023, *P* < 0.001), lower educational level (OR = 0.934, *P* < 0.001), head tremor (OR = 1.457, *P* <0.001), and higher TETRAS-II scores (OR = 1.134, *P* < 0.001) were significantly associated with ET plus (Table 2).

### AAO affect clinical heterogeneity of ET

In total, 51.0% of ET patients with AAO < 60 years had familial ET, while only 40.4% of late-onset patients (AAO≥ 60 years) had a positive family history (X^2^ = 7.859, *P* = 0.005). The late-onset ET patients had a lower proportion of self-reported alcohol sensitivity than those with AAO < 60 years (45.8% vs. 20.7%; X^2^ = 17.871, *P <* 0.001). Logistic regression indicated that facial tremor (OR = 2.415, *P* < 0.001), voice tremor (OR = 1.839, *P* = 0.001), lower limb tremor (OR = 1.565, *P* = 0.015), mild cognitive impairment (OR = 3.038, *P* < 0.001), impaired tandem gait (OR = 2.301, *P* < 0.001), and questionable myotonia (OR = 2.504, *P* = 0.010) were significantly associated with late-onset ET after adjusting for sex and disease duration. Late-onset ET patients were more likely to present with kinetic tremor (OR = 2.937, *P* = 0.009). Patients with late-onset ET also had higher TETRAS-I, TETRAS-II, and NMSS scores than those with AAO<60 years (all *P* < 0.001) (Table 3).

**Table 4:** Comparison of demographic and clinical characteristics between elderly ET patients and younger patients.

Items	Younger than 65 years of age (n = 759)	Aged 65 years or older (n = 401)	*P*-value
**Age (y)**	46.57±13.53	70.87±5.49	**<0.001**
**AAO (y)**	36.59±14.57	58.43±12.37	**<0.001**
**Sex (male %)**	402 (53.0%)	185 (46.1%)	**0.027**
**Duration (y)**	9.98±8.54	12.439±11.49	**0.040**
**Family history (%)**	375 (49.4%)	194 (48.4%)	0.739
**Alcohol sensitivity (%)**	169/373 (45.3%)	54/159 (34.0%)	**0.015**
**Tremor distribution**			
**Head (%)**	232 (30.6%)	138 (34.4%)	0.269^#^
**Face (%)**	139 (18.3%)	128 (31.9%)	**<0.001^#^**
**Voice (%)**	175 (23.1%)	124 (30.9%)	**0.006^#^**
**Upper limbs (%)**	759(100%)	401 (100%)	-
**Lower limbs (%)**	213 (28.1%)	117 (29.2%)	0.689^#^
**Tremor types**			
**Posture tremor (%)**	759(100%)	401 (100%)	-
**Kinetic tremor (%)**	719 (94.7%)	330 (97.3%)	0.050^#^
**Intention tremor (%)**	260 (35.3%)	160 (39.9%)	0.057^#^
**Tremor severity**			
**TETRAS-I**	11.52±9.01	17.11±10.30	**<0.001^#^**
**TETRAS-II**	16.85±7.79	20.66±8.60	**<0.001^#^**
**Tremor asymmetry**	246 (32.4%)	155 (38.7%)	0.325^#^
**ET plus**	346 (44.6%)	268 (66.8%)	**<0.001^#^**
**Mild cognitive impairment**	146(19.2%)	150 (37.4%)	**0.002^#^**
**Rest tremor**	128(16.9%)	90 (22.4%)	**0.021^#^**
**Questionable dystonic posturing**	116(15.3%)	73 (18.2%)	0.289^#^
**Impaired tandem gait**	71(9.4%)	91 (22.7%)	**<0.001^#^**
**Questionable myotonia**	23(3.0%)	29 (7.2%)	**0.003^#^**
**NMSS**	10.76±14.00	16.04±16.57	**<0.001^#^**

Data for continuous variables are presented as mean ± standard deviation. *P* value ^#^ is from linear or logistic regression and adjusted with variables of sex and AAO. Values in bold refer to statistically significant differences (*P* < 0.05). Abbreviations: ET, essential tremor; y, years; AAO, age at onset; TETRAS, Tremor Research Group Essential Tremor Rating Assessment Scale; NMSS, Non-Motor Symptoms Scale.

### Age affects clinical heterogeneity of ET

A total of 401 patients (34.6%) were elderly (aged 65 years or older) and 759 (65.4%) were younger than 65 years of age. The proportion of males was higher in elderly ET patients than in others (53.0% vs. 46.1%; X^2^ = 4.896, *P* = 0.027). The mean AAO of the elderly ET patients was higher than that of those aged younger than 65 years (Mann-Whitney U test, *P* < 0.001). Elderly ET patients had a higher frequency of self-reported alcohol sensitivity than patients younger than 65 years (45.3% vs. 34.0%; X^2^ = 5.894, *P* = 0.015). After adjusting for sex and AAO, logistic regression indicated that facial tremor (OR = 2.030, *P* < 0.001), voice tremor (OR = 1.467, *P* = 0.006), mild cognitive impairment (OR = 1.749, *P* = 0.001), impaired tandem gait (OR = 2.761, *P* < 0.001), rest tremor (OR = 1.427, *P* = 0.021), and questionable myotonia (OR = 2.459, *P* = 0.003) were significantly associated with ET in the elderly. Elderly ET patients had higher TETRAS-I, TETRAS-II, and NMSS scores (all *P* < 0.001).

### Sex affects clinical heterogeneity of ET

The clinical characteristics of ET showed conspicuous differences between the male and female patients (Table 5). Female patients were older with respect to both mean age upon enrollment (57.37±13.91 years vs 52.63±17.94 years, Mann-Whitney U test, *P* < 0.001) and mean AAO (47.01±15.77 years vs 41.34±18.28 years, Mann-Whitney U test, *P* < 0.001), and they had lower alcohol sensitivity (23.1% vs 54.4%; X^2^ = 51.187, *P* < 0.001). Disease duration showed no significant difference between the different sex groups (Mann-Whitney U test, *P* = 0.051). After adjusting for age and AAO, logistic regression indicated that the female sex was associated with midline tremors, including head tremors (OR = 1.556, *P* = 0.001), facial tremors (OR = 1.765, *P* < 0.001), and voice tremors (OR = 1.317, *P* = 0.044). Mild cognitive impairment (OR = 1.433, *P* = 0.010), questionable dystonic posturing (OR = 1.680, *P* = 0.002), impaired tandem gait (OR = 1.735, *P* = 0.002), and questionable myotonia (OR = 2.559, *P* = 0.004) were significantly more prevalent in female patients. After adjusting for age and AAO, the TETRAS-I and TETRAS-II scores (all *P*<0.001) were significantly higher in male patients than those in females. Sex was not correlated with tremor type, asymmetry, or severity. Regarding non-motor symptoms, female patients had significantly higher NMSS scores (*P* = 0.002).

**Table 5:** Comparison of demographic and clinical characteristics between male and female patients.

Items	Male (n=587)	Female (n=573)	*P*-value
**Age (y)**	52.63±17.94	57.37±13.91	**<0.001**
**AAO (y)**	41.34±18.28	47.01±15.77	**<0.001**
**Duration (y)**	11.29±9.759	10.36±9.671	0.103
**Family history (%)**	292 (49.7%)	277 (48.3%)	0.633
**Alcohol sensitivity (%)**	174/320 (54.4%)	49/212 (23.1%)	**<0.001**
**Tremor distribution**			
**Head (%)**	158 (26.9%)	212 (37.0%)	**0.001^#^**
**Face (%)**	102 (17.4%)	165 (28.8%)	**<0.001^#^**
**Voice (%)**	134 (22.8%)	165 (28.8%)	**0.046^#^**
**Upper limbs (%)**	587 (100%)	573 (100%)	-
**Lower limbs (%)**	177 (30.2%)	153 (26.7%)	0.212^#^
**Tremor types**			
**Posture tremor (%)**	587 (100%)	573 (100%)	-
**Kinetic tremor (%)**	563 (95.9%)	546 (95.3%)	0.487^#^
**Intention tremor (%)**	225 (38.3%)	195 (34.0%)	**0.072^#^**
**Tremor severity**			
**TETRAS-I**	13.85±9.62	13.05±10.04	**0.008^#^**
**TETRAS-II**	18.46±8.01	17.86±8.54	**0.031^#^**
**Tremor asymmetry**	202 (34.4%)	199 (34.7%)	0.790^#^
**ET plus**	264(45.0%)	350(61.1%)	**<0.001^#^**
**Mild cognitive impairment**	123(21.0%)	173 (30.2%)	**0.005^#^**
**Rest tremor**	102(17.3%)	116 (20.2%)	0.291^#^
**Questionable dystonic posturing**	74(12.6%)	115 (20.1%)	**0.002^#^**
**Impaired tandem gait**	59(10.1%)	103 (18.0%)	**0.001^#^**
**Questionable myotonia**	14(2.4%)	38 (6.6%)	**0.004^#^**
**NMSS**	10.99±14.42	14.47±15.70	**0.002^#^**

Data for continuous variables are presented as mean ± standard deviation. *P* value ^#^ is from linear or logistic regression and adjusted with variables of age and AAO. Values in bold refer to statistically significant differences (*P* < 0.05). ET, essential tremor; y, years; AAO, age at onset; TETRAS, Tremor Research Group Essential Tremor Rating Assessment Scale; NMSS, Non-Motor Symptoms Scale.

## DISCUSSION

The classic diagnosis of ET was previously made based on diagnostic criteria established in 2000 [31]. Nevertheless, as a result of the growing knowledge on ET, a consensus statement was issued by the IPMDS in 2018, facilitating more in-depth and detailed phenotyping of patients with ET. To keep up with this new update, numerous studies on ET retrospectively reclassified ET patients who had been evaluated before 2018 according to the consensus statement [11-14]. However, these retrospective reevaluations were subject to diagnostic bias. To conduct an unbiased study that evaluates the clinicodemographic characteristics of ET patients and explores the possible factors related to ET plus in detail, the China Essential Tremor Alliance launched a large multicenter observational study of ET in the context of the new classification criteria, namely NSETP-China. Our study showed that ET plus was more common than pure ET and that patients from the two groups demonstrated several phenotypic differences. These findings were consistent with those of the previous studies [6,8,10,11,13,14]. In addition, ET plus was found to be more common in females than in males, and ET plus patients were generally older and had a later onset, longer disease duration, worse tremors, and more severe non-motor symptoms.

ET plus may not be a monosymptomatic disorder, as it may involve cognitive functions and the cerebellum. The most prevalent soft neurological signs were mild cognitive impairment, resting tremor, questionable dystonic posturing, impaired tandem gait, and questionable myotonia. ET plus patients had a higher prevalence of midline tremors (including head, face, and voice tremors) and lower limb tremors. The current study also found that female sex, older age, lower education level, head tremor, lower limb tremor, and higher attention/memory subscores were significantly associated with ET plus. Furthermore, the female sex was a significant contributor to ET. Sex differences in ET plus may arise from the interaction between hormones and the environment. Several studies have shown that upper limb dystonia and cognitive impairment are more common in females [32,33]. Head tremors independently increased the risk of ET plus. A longitudinal study also showed that head tremors are strongly associated with dystonic postures of the neck [34]. Meanwhile, midline tremors might be related to severe cerebellar dysfunction, especially in the cerebellar posterior and vermis [35,36]. Midline tremors are more likely to manifest as an impaired tandem gait [37]. Consistent with these findings, the current study found that head tremors were associated with ET plus. A higher education level could delay cognitive decline and reduce the risk of mild cognitive impairment [38,39]. A lower educational level might be an important predictive factor for mild cognitive impairment in patients with ET.

Moreover, the current study found that ET plus patients were generally older and had a longer disease duration than ET patients, suggesting that ET plus may represent a relatively late feature in long-standing ET patients. It is well known that alcohol responsiveness is a common feature of ET [40,41]. Most patients with a history of drinking experienced an improvement in their tremors with alcohol consumption, and there was no significant difference between ET plus and ET patients. Genetics plays a significant role in the pathogenesis of ET [42,43]. However, the prevalence of positive family history was not significantly different between the groups in the current study. This suggests that neurological soft signs do not interact with a positive family history or alcohol responsiveness. We speculated that ET and ET plus likely represent different stages of a single disease, and some ET patients might progress to developing ET plus over time. A recent neuropathological study indicated that there were no pathological differences in the cerebellar cortex between ET plus and ET patients [7,17]. Several studies have also supported the hypothesis that ET plus and ET may not represent distinct clinicopathological entities [17,44-46]. Neurological soft signs observed in some ET plus patients could be the physiological effects of aging superimposed on a pure ET. To verify this hypothesis and monitor disease progression, both pure ET and ET plus patients in NSETP-China will be scheduled to undergo a 5-year longitudinal clinical evaluation.

Meanwhile, we also noticed a bimodal AAO distribution in pure ET patients in their 2^nd^ and 5^th^ decades and only one peak in ET plus patients in their 6^th^ decade. The AAO of ET plus patients tended to be later than that of the ET patients. A previous study also showed that ET plus patients had a shorter disease duration than pure ET patients [20]. This suggests that ET plus may not be a disease stage of ET but a disease entity. Furthermore, our study demonstrated that clinical manifestations differed between ET patients with AAO before and after 60 years of age, similar to a previous report [38]. Tremor and non-motor symptoms were more severe in patients with late-onset ET. Compared with ET patients with AAO < 60 years, those with AAO ≥ 60 years had a higher prevalence of voice tremors. As expected, and indicated by previous studies [5,47,48], patients with AAO aged ≥ 60 years were more likely to have a mild cognitive impairment, impaired tandem gait, and questionable myotonia. Our study further underscores the differences between the two AAO groups. Questionable dystonic posturing and resting tremors were not associated with AAO. It also had been demonstrated that ET patients with AAO > 65 years were more likely to have mild cognitive impairment than age-matched healthy controls, whereas ET patients with AAO < 65 years were no more likely to have mild cognitive impairment [49,50]. Longitudinal studies also showed that the rate of cognitive decline seemed to be faster in ET patients with AAO > 65 years than in healthy controls. Therefore, late-onset ET is associated with mild cognitive impairments. Elderly individuals were far more likely to have subclinical neurological comorbidities than young patients [51]. These results indicate that, in some cases, late-onset ET patients were more likely to be diagnosed with ET plus at onset, rather than a more advanced stage of ET. Concurrently, the current study found a predominance of female patients in the ET plus group. There was an obvious sex difference between ET and ET plus. ET plus was associated with midline tremors. Therefore, it is also possible that ET plus may represent a group of different entities.

In addition to the soft signs mentioned in our study, ET patients experience a myriad of additional neurological features, such as hearing impairment, peripheral neuropathy, anxiety, depression, apathy, and sleep disturbances [48,52]. There was no consensus on which additional signs were acceptable within the definition of soft signs. According to the 2018 consensus, the soft neurological signs in our study were restricted to those listed in the methods. Patients with ET with neuropsychiatric features were not diagnosed with ET plus. Long-term follow-up is necessary for such patients. In our study, non-motor symptoms were present in 61.4% of patients with ET plus and only 46.3% of patients with pure ET. Sleep disturbances/fatigue and mood disturbances/apathy are among the most common non-motor symptoms in patients with ET. Non-motor symptoms were more severe in ET plus patients than in those with pure ET. The frequency of all non-motor symptoms, except sexual function, was significantly higher in the ET plus group than in the pure ET group. Neurological soft signs may have more negative effects on non-motor symptoms in ET patients. A previous study found that the mean total NMSS score of patients with PD in China was 36.06[53]. This means that ET patients have milder non-motor symptoms than PD patients.

Our study had some limitations. This is a cross-sectional study and barely reflects the dynamics of disease progression. It remains unclear whether pure ET and ET plus are merely different stages of a single disease or represent distinct disease entities. Further longitudinal follow-up studies are required to confirm these findings. However, a 5-year longitudinal clinical evaluation will address this shortcoming. The fact that participants in NSETP-China were recruited from different clinical sites is prone to interviewer bias. Nevertheless, all researchers had undergone standardized and unified training by the NSETP-China Steering Committee to maintain data consistency.

## Conclusions

Old age and the female sex may contribute to the development of ET plus. However, resting tremors were independent of age and sex. Pure ET showed a bimodal distribution for AAO, whereas ET plus showed a unimodal distribution. Patients with late-onset ET were more likely to present with soft neurological signs and more severe symptoms than patients with AAO aged < 60 years. It remains unclear whether pure ET and ET plus are merely different stages of a single disease or represent distinct disease entities.
